# Genome-derived insights into the biology of the hepatotoxic bloom-forming cyanobacterium *Anabaena* sp. strain 90

**DOI:** 10.1186/1471-2164-13-613

**Published:** 2012-11-13

**Authors:** Hao Wang, Kaarina Sivonen, Leo Rouhiainen, David P Fewer, Christina Lyra, Anne Rantala-Ylinen, Johanna Vestola, Jouni Jokela, Kaisa Rantasärkkä, Zhijie Li, Bin Liu

**Affiliations:** 1 Department of Food and Environmental Sciences, University of Helsinki, Helsinki, FIN-00014, Finland; 2 BGI LifeTech Co., Ltd., Beijing, China; 3 Center of Systematic Genomics, Xinjiang Institute of Ecology and Geography/Institute of Oceanology, Chinese Academy of Sciences, 818 South Beijing Road Urumqi, 830011, Xinjiang, China

**Keywords:** Cyanobacteria, *Anabaena*, Mobile genetic elements, Insertion sequences, Biosynthetic gene clusters, Restriction-modification system, *nifH* excision element

## Abstract

**Background:**

Cyanobacteria can form massive toxic blooms in fresh and brackish bodies of water and are frequently responsible for the poisoning of animals and pose a health risk for humans. *Anabaena* is a genus of filamentous diazotrophic cyanobacteria commonly implicated as a toxin producer in blooms in aquatic ecosystems throughout the world. The biology of bloom-forming cyanobacteria is poorly understood at the genome level.

**Results:**

Here, we report the complete sequence and comprehensive annotation of the bloom-forming *Anabaena* sp. strain 90 genome. It comprises two circular chromosomes and three plasmids with a total size of 5.3 Mb, encoding a total of 4,738 genes. The genome is replete with mobile genetic elements. Detailed manual annotation demonstrated that almost 5% of the gene repertoire consists of pseudogenes. A further 5% of the genome is dedicated to the synthesis of small peptides that are the products of both ribosomal and nonribosomal biosynthetic pathways. Inactivation of the hassallidin (an antifungal cyclic peptide) biosynthetic gene cluster through a deletion event and a natural mutation of the buoyancy-permitting *gvpG* gas vesicle gene were documented. The genome contains a large number of genes encoding restriction-modification systems. Two novel excision elements were found in the *nifH* gene that is required for nitrogen fixation.

**Conclusions:**

Genome analysis demonstrated that this strain invests heavily in the production of bioactive compounds and restriction-modification systems. This well-annotated genome provides a platform for future studies on the ecology and biology of these important bloom-forming cyanobacteria.

## Background

Cyanobacteria are evolutionarily important prokaryotic organisms that created the oxygenic atmosphere on Earth via oxygenic photosynthesis and were the progenitors of chloroplasts in eukaryotic algae and plants [1]. Cyanobacteria often dominate phytoplankton as surface scum in freshwater lakes and brackish water during the summer months [2]. A small number of cyanobacterial genera are typically involved in bloom formation [2]. Gas vesicles are common in planktonic cyanobacteria and allow the organisms to regulate their buoyancy [3]. Bloom-forming cyanobacteria produce an array of potent hepatotoxins and neurotoxins [4]. Microcystins are commonly reported hepatotoxic heptapeptides that inhibit eukaryotic protein phosphatases 1 and 2A [2]. Toxic blooms are responsible for the toxicoses of wild and domestic animals [5] and are a health risk for humans through the consumption or recreational use of water [6].

*Anabaena* is a genus of filamentous nitrogen-fixing cyanobacteria [7] that is especially common in aquatic environments, both in fresh and brackish waters worldwide [8,9]. Nitrogen fixation occurs in specialized cells called heterocysts that differentiate from the vegetative cells [10]. This property combined with photosynthesis makes *Anabaena* cyanobacteria autotrophic organisms that are able to live in a wide range of environments. Strains of the planktonic *Anabaena* genus are some of the most common cyanobacteria capable of forming blooms [4]. Blooms of *Anabaena* are a serious health risk, due to the production of a range of toxins such as microcystins, anatoxins and saxitoxins [4,11].

Cyanobacteria, including *Anabaena*, are prolific sources of natural products, many of which have biotechnological and biomedical importance [11]. In recent years, many new compounds and their biosynthetic pathways have been discovered [11,12]. The cyanobacterial hepatotoxins, microcystins and nodularins, are the end products of nonribosomal biosynthetic pathways [13]. Recently, it has been shown that cyanobacteria also use diverse ribosomally encoded pathways for the production of small linear and cyclic peptides [14,15]. To understand the role of these bioactive compounds in cyanobacteria, as well as the biotechnological exploitation of the biosynthetic machinery used to assemble them, more information on the regulation and association of these biosynthetic pathways with other metabolic processes is needed.

Only a small number of genomes for planktonic bloom-forming cyanobacteria are known, including *Microcystis aeruginosa* PCC 7806 and NIES-843 [16,17], *Planktothrix rubescens* NIVA CYA 98 [18], *Cylindrospermopsis raciborskii* CS-505 and *Raphidiopsis brookii* D9 [19], which produce microcystins, cylindrospermopsin or saxitoxin. Here, we present the complete genome of *Anabaena* sp. strain 90, a bloom-forming, microcystin-producing strain originating from a freshwater lake in Finland.

## Results

### Genome overview

**Figure 1:**
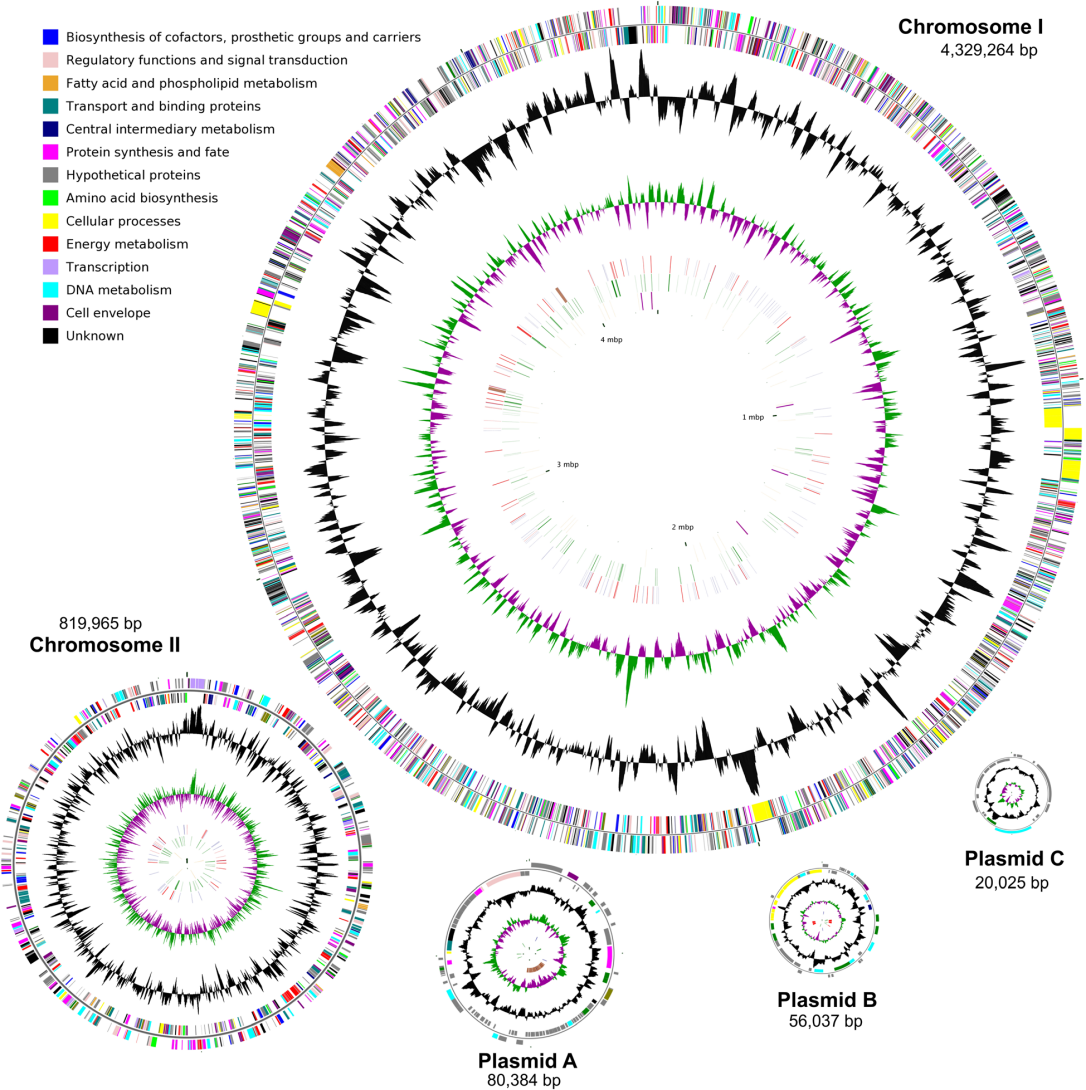
**Circular demonstration of the two chromosomes and three plasmids of the *Anabaena *sp. 90 genome.** The outermost and second circles of all replicons indicate genes in forward and reverse orientation colour-coded by their role categories as shown. The third circles of all replicons show G+C content in black and the fourth circles show the G+C skew in green (+) and purple (−). The fifth circles show the insertion sequences in red, putative prophage remnants in brown and MITEs in blue. The sixth circles show the pseudogenes with disrupted ORFs in green. The innermost circles of chromosome I and II show the rRNA genes in purple and tRNA genes in orange. The circular sizes of the replicons are not proportional to the actual sizes

The *Anabaena* sp. 90 genome was assembled with Sanger reads that were sequenced from libraries with different size inserts (2, 6 and 40 kb) and amounted to a 12.5 X depth of coverage. The remaining physical gaps that were derived from the unclonable regions were linked through combinatorial multiplex PCR screening of primers designed from the contig ends. The genome consists of five circular replicons, two chromosomes and three plasmids (Figure 1, Table 1). The total size of the genome amounted to 5,305,675 bp with an average G+C content of 38.1%. The quality of the genome sequence was very high and the estimated overall sequence error of the genome was 0.12 bp (Table 1). A total of 4,738 ORFs were annotated with putative functions assigned to 2,954 (62.35%) ORFs from manual annotation. The remaining 1,784 (37.65%) were assigned as hypothetical ORFs (see Additional file 1: Table S1). They were further subgrouped as 480 (10.13%) conserved hypothetical proteins that have more than 30 counterparts in other bacterial genomes, and 205 (4.33%) unique proteins that have no full-length counterparts (see Methods). In addition, there are 1099 (23.19%) hypothetical ORFs that lie in between, having few counterparts in other genomes. Five rRNA operons were identified and dispersed throughout chromosome I, two in the leading and three in the lagging strand (Table 1). They have nearly identical rRNA genes. Sequence variations in the spacer regions separate them into two groups. Two operons with consecutive tRNAs organized as *16S*-*trnI-trnA*-*23S*-*5S* form one group. Members in another group have no tRNA genes. A total of 44 tRNAs were distributed over both chromosome I (40) and II (4).

**Table 1:** **Summary of the *Anabaena *sp. 90 genome**

	**Length (bp)**	* Errors/Mb	**GC (%)**	**# ORFs**					**# RNAs**	
				**Total**	**Hypothetical**	**(%)**	**Pseudogene**	**(%)**	**rRNA**	**tRNA**
Chromosome I	4,329,264	0.02	38.1	3,817	1,414	(37.04)	169	(4.43)	15	40
Chromosome II	819,965	0.03	38.2	755	256	(33.91)	49	(6.49)	0	4
Plasmid A	80,384	0.03	37.2	93	71	(76.34)	5	(5.38)	0	0
Plasmid B	56,037	0.01	37.3	51	23	(45.10)	4	(7.84)	0	0
Plasmid C	20,025	0	37.42	22	20	(90.91)	0	(0)	0	0
**Total**	**5,305,675**	**0.022**	**38.1**	**4,738**	**1,784**	**(37.65)**	**227**	**(4.79)**	**15**	**44**

* Each called read base was assigned a Phred score that is logarithmically related to its error probabilities [76,77]. The error rate of each replicon was determined as the sum of the error probabilities over all assembled bases.

Gene composition between the chromosomes and plasmids is highly biased. All enzymes of essential biological pathways, such as photosynthesis, are encoded in the two chromosomes. The three plasmids mostly encode integrases, recombinases, transposases, phage-related proteins and a high percentage of hypothetical proteins (Table 1).

### Prophage remnants

**Figure 2:**
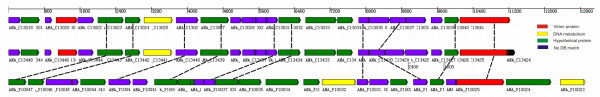
**Organization of genes in the regions of the three putative prophage remnants.** The remnants of the three putative prophages were found in the presence of the phage genes and highly compact organization. Two of the putative prophage remnants (~11 kb) on chromosome I share high sequence similarity (93.5%) and are located 500 kb apart. The third region of the prophage remnants (~13 kb) was located in plasmid A. Genes identified with BLASTp as having similarity to known phage genes are marked in red, DNA metabolism genes in yellow, hypothetical genes in green, and the novel genes without database counterpart in purple. The homologue genes shared among the three prophages were indicated by broken lines

Remnants of three putative prophages were found, due to the presence of phage genes with a highly compact organization (Figure 2). However, ORFs encoding phage terminases and capsid protein-encoding genes were absent. Two of the putative prophage remnants are 11 kb in size and display high sequence similarity (93.5%) but are located 500 kb apart in chromosome I. The third prophage remnant is located in plasmid A with a slightly larger size (13 kb). These three prophage remnants have similar gene organization and share homologous genes (Figure 2), suggesting a close relationship between them. This indicates that *Anabaena* sp. 90 may have been a lysogen at some point in the past. Prophages are frequently inserted into tRNA genes [20]. A truncated C-terminal tRNA (Thr) gene was found 2.2 kb downstream of the first prophage remant in chromosome I. In addition, there is a 14-kb gene cluster containing clustered regularly interspaced short palindromic repeats (CRISPRs) and associated *cas* genes found in this genome in plasmid B. It was classified as subtype I-D, according to recent studies [21,22]. This gene cluster may provide acquired phage resistance for the strain. 

### Pseudogenes and transposable elements

**Figure 3:**
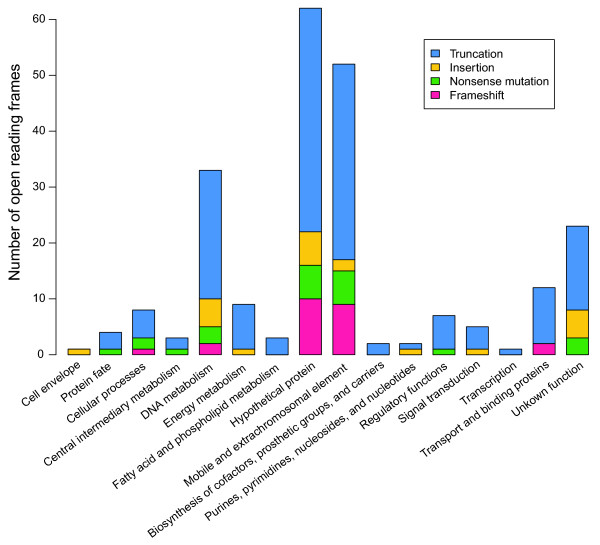
**Compositional distribution of 227 pseudogenes among different functional categories.** Four types of pseudogenes were found and colour-coded as truncation in blue, insertion in orange, nonsense mutation (premature stop codon) in green and frameshift in pink. The functional category of the Comprehensive Microbial Resource database was applied [23]

Surprisingly, a total of 227 pseudogenes were discovered across the genome through inspection of the disrupted ORFs (see Additional file 1: Table S2). More than half of the pseudogenes (114) were assigned to the ‘hypothetical protein’ and ‘mobile and extrachromosomal element’ categories, based on the Comprehensive Microbial Resource database [23] (Figure 3). There are 33 pseudogenes related to DNA metabolism, 13 of which were predicted with functions involved in DNA restriction and modification. Most of the pseudogenes (69.6%) were derived from truncation of the ORFs. Other types of pseudogenes that were found either contain frame shifts (10.6%), nonsense mutations (10.1%) or insertions (9.7%). The distribution of these pseudogenes overlaps that of the mobile genetic elements, especially insertion sequences (ISs) (Figure 1). 

In all, 81 complete and partial ISs, with a total size of 96.6 kb, were annotated from the *Anabaena* sp. 90 genome (see Additional file 1: Table S3). Sixty-five of these ISs were classified into 22 subfamilies, according to the ISfinder database [24], 18 of which occurred in two to seven copies (Table 2). Sixteen of the elements could not be assigned to a family in the ISfinder database. Pseudogenes with disrupted ORFs were often found adjacent to ISs (Figure 1), where 46 disrupted transposases were also discovered (Figure 3, see Additional file 1: Table S2). IS transposition disrupted not only host-specific ORFs but also the ORFs of other IS element transposases (Figure 3). 

**Table 2:** **Categorized IS subfamilies in the *Anabaena *sp. 90 genome**

**Name**	**Family and Subgroup**	**Size (bp)**	**Number**
ISAsp2	ISAs1	1627	6
ISAsp3	IS4 ssgr ISPepr1	1462	5
ISAsp4	IS1380	1005	5
ISAsp5	IS1634	2038	6
ISAsp6	IS607	1353	3
ISAsp7	IS607	862	1
ISAsp8	IS607	1242	2
ISAsp9	IS607	1575	7
ISAsp10	IS4 ssgr IS10	1270	2
ISAsp11	IS200/IS605 ssgr IS1341	1790	4
ISAsp12	IS200/IS605 ssgr IS1341	1685	3
ISAsp13	IS200/IS605	1607	2
ISAsp14	IS200/IS605	1798	3
ISAsp15	IS607	1660	2
ISAsp16	IS630	1228	3*
ISAsp17	IS200/IS605 ssgr IS1341	1060	1
ISAsp18	IS200/IS605 ssgr IS1341	1223	1
ISAsp19	IS200/IS605 ssgr IS608	1652	2
ISAsp20	IS200/IS605 ssgr IS200	682	1
ISAsp21	IS5 ssgr IS5	1275	2
ISAsp22	IS607	1866	2
ISAsp23	IS5 ssgr IS1031	413	2*

* all members of the subfamily are disrupted.

**Figure 4:**
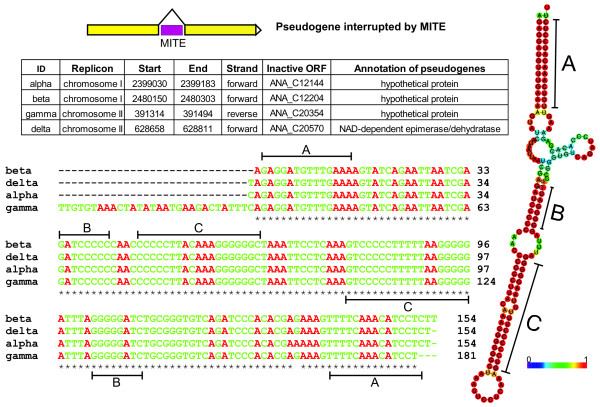
**Sample group of MITEs causing pseudogenes in the *Anabaena *sp. 90 genome.** Four MITEs in group 1 that interruptedly caused pseudogenes were demonstrated. The secondary structure was predicted with the sequence data using RNAfold [84], while similarity of the paired bases was indicated by the colour scheme shown. Three sets of inverted repeat regions (A, B and C) were located from the secondary structure of the sequences, and labelled in the multisequence alignment

In addition, a total of 147 complete or partial miniature inverted-repeat transposable elements (MITEs) were located in the genome. Their lengths ranged from 77 to 541 bp (see Additional file 1: Table S4). These small genetic elements are usually present in multiple copies and are characterized by terminal inverted repeat regions [25]. The MITEs identified in this study could be grouped into type II MITEs. A total of 132 MITEs were distributed in intergenic regions, while 15 were found within disrupted chromosomal ORFs and led to the prediction of pseudogenes (Figure 4). 

### Bioactive peptide synthesis

**Figure 5:**
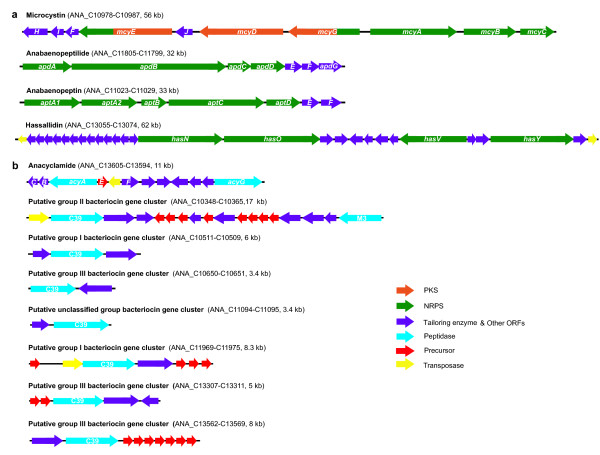
**Biosynthetic gene clusters of nonribosomal and ribosomal peptides in the *Anabaena *sp. 90 genome.** The genetic organization of the biosynthetic gene clusters for producing nonribosomal (**a**) and ribosomal (**b**) peptides. The putative bacteriocin gene clusters were classified according to methods described previously [15]

*Anabaena* sp. 90 produces many bioactive peptides by nonribosomal or ribosomal pathways. In addition to the previously identified nonribosomal biosynthetic gene clusters for anabaenopeptilides [26], anabaenopeptins [27] and microcystins [28], a large gene cluster responsible for production of glycolipopeptides (hassallidins) was found (Figure 5). In addition to an anacyclamide-encoding cyanobactin gene cluster [29], seven putative bacteriocin gene clusters were also discovered (Figure 5). All these biosynthetic gene clusters were located in chromosome I. Together they amount to a total of ~250 kb, thus at least 5% of the genome is dedicated to the production of bioactive peptides. 

**Figure 6:**
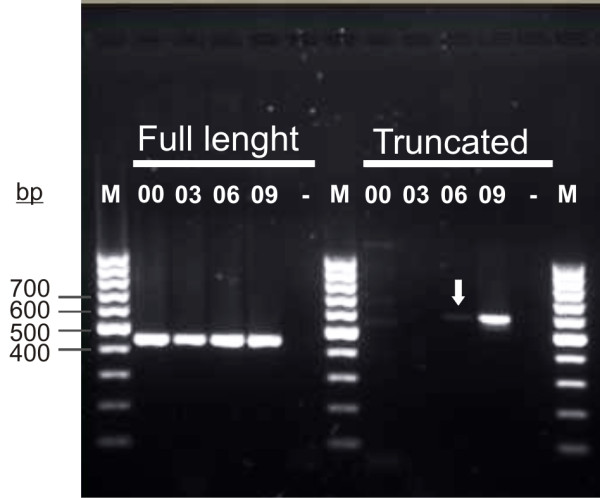
**PCR screening of the deletion in the *hasV *gene with *Anabaena *sp. 90 cells originating from different years.** PCR analysis of genomic DNA demonstrates occurrences of the deletion in *hasV* in 2000, 2003, 2006 and 2009. PCRs with primers across the site of deletion show that the deletion appeared in 2006 (arrow). Recent cultures of *Anabaena* sp. 90 carry a deletion in *hasV* and this strain no longer produces hassallidin

**Figure 7:**
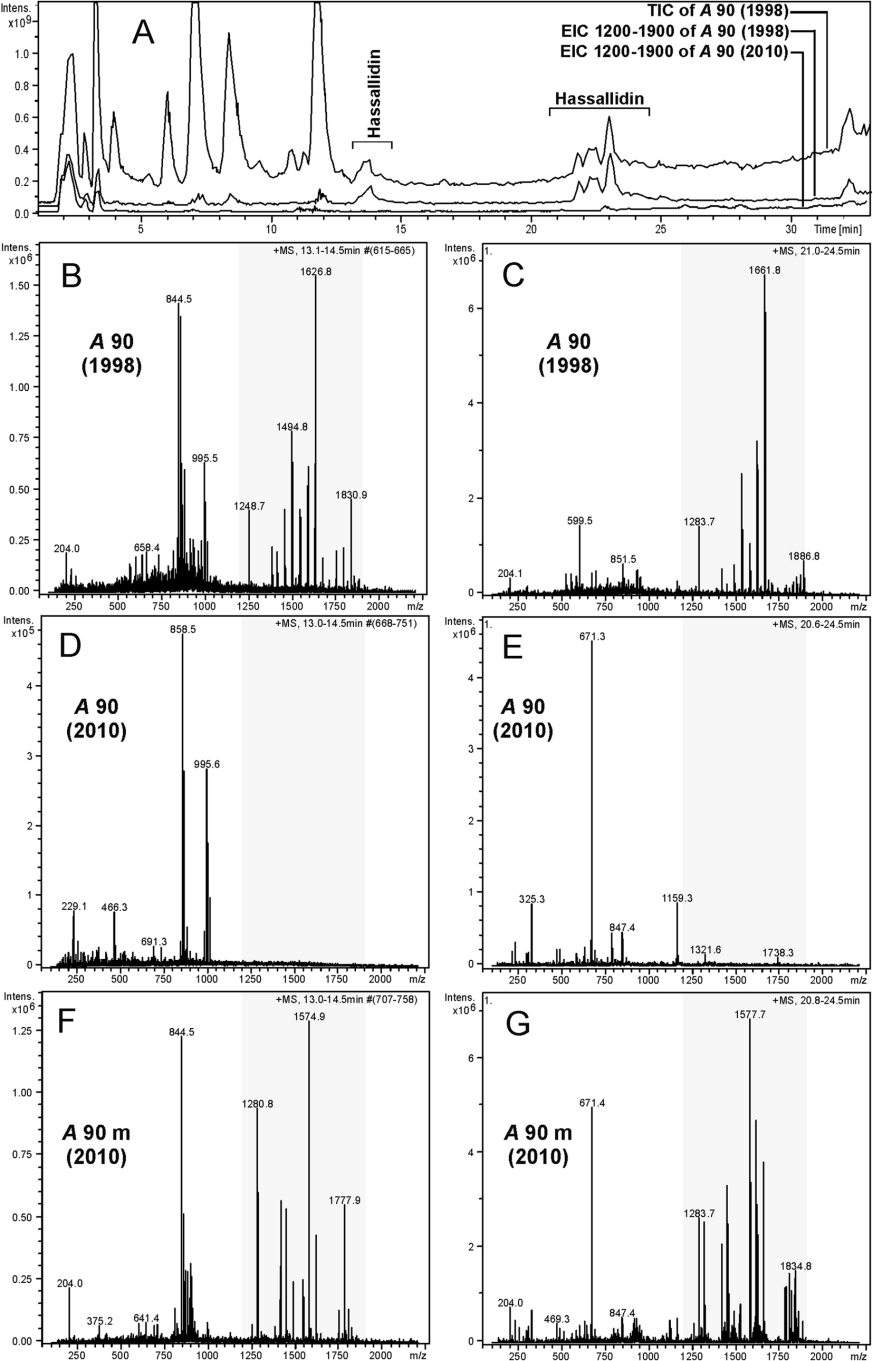
**Chromatograms and mass spectra show the presence and absence of hassallidin variants in *Anabaena *sp. 90. A**: Total ion chromatogram (TIC) of *Anabaena* sp. 90, originating in 1998, and extracted ion chromatograms (EICs) of *Anabaena* sp. 90, originating from 1998 and 2010, show the elution of ions with *m/z* 1200 – 1900, representing the mass range of hassallidin ions. Two eluting time ranges of the hassallidin variants are marked in the chromatograms. **B**-**G**: Average mass spectra from the first (**B**, **D**, **F**) and the second (**C**, **E**, **G**) time range of the chromatograms in which the hassallidin variants were eluting. The grey areas show the hassallidin ions in the spectra. **B**, **C**: Spectra from *Anabaena* sp. 90 originating from 1998. **D**, **E**: Spectra from *Anabaena* sp. 90 originating from 2010. **F**, **G**: Spectra from *Anabaena* sp. 90 anabaenopeptilide minus mutant originating from 2010

The hassallidin biosynthetic gene cluster is the largest gene cluster in *Anabaena* sp. 90. It encodes four nonribosomal peptide synthetase enzymes that catalyse the incorporation of amino acid residues into cyclic hassallidin peptides (Vestola et al., unpublished data). Surprisingly, the genome assembly revealed two different contigs within the same sequence, which led us to the discovery of a 526-bp deletion in the peptide synthetase gene *hasV* (ANA_C13069). The deletion introduced a frameshift and rendered the gene cluster nonfunctional. In PCRs performed with *Anabaena* sp. 90 DNA originating from the years 1998–2009, the deletion first appeared around 2003–2006, but had not fully segregated by 2009 (Figure 6). Due to the deletion, hassallidins can no longer be detected in current cultures of *Anabaena* sp. 90. However, hassallidins could still be identified from cells extracted prior to the deletion in 1998 and from an anabaenopeptilide synthetase gene mutant (*apdA*^*-*^) strain of *Anabaena* sp. 90 constructed in 1999 [26] (Figure 7). 

### Restriction-modification systems

**Figure 8:**
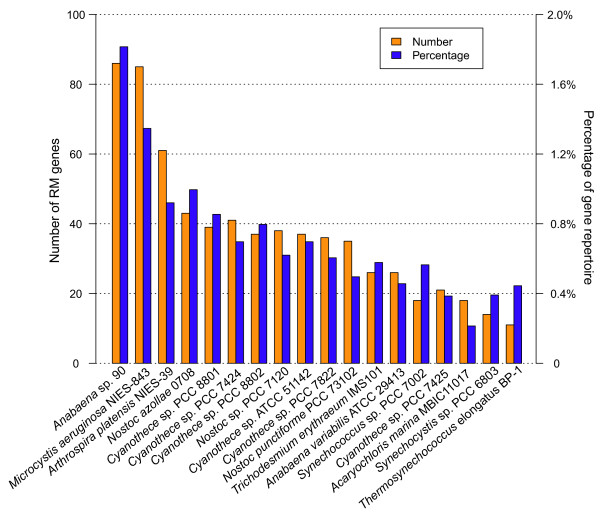
**Number and percentage of RM genes found in the *Anabaena *sp. 90 and other cyanobacterial genomes.** The RM genes in other cyanobacterial genomes were collected from the REBASE Genomes [85]

The *Anabaena* sp. 90 genome contains a very high number (88) of restriction-modification (RM) system-related ORFs, amounting to 1.8% of the gene repertoire. Another bloom-forming cyanobacterium, *Microcystis aeruginosa* NIES-843, had nearly the same number of RM system-related ORFs, but other cyanobacteria had lower numbers of RM genes (Figure 8). There are 31 Type I, II, III and IV RM systems, constituting 56 chromosomal ORFs in *Anabaena* sp. 90 (see Additional file 1: Table S5). The majority (22 out of 31) were categorized as type II systems. In addition, there are 14 separate restriction enzymes and 18 unaccompanied DNA methyltransferases. However, 13 of them are pseudogenes with disrupted ORFs, including five enzymes in type I, II and III RM systems, three restriction enzymes and five DNA methylases.

### Photosynthesis

The photosynthetic gene clusters in the genome retain the conserved organizational pattern found in other cyanobacteria [30]. A quarter of the photosynthetic genes are distributed in chromosome II, such as *psbA*, *psbB*, and operons of *atpIHGFDAC* encoding ATP synthase, *petBD* cytochrome b6/f complex and *apcABC* light antenna. The rest of the photosynthetic genes were found in chromosome I.

### Nitrogen fixation

**Figure 9:**
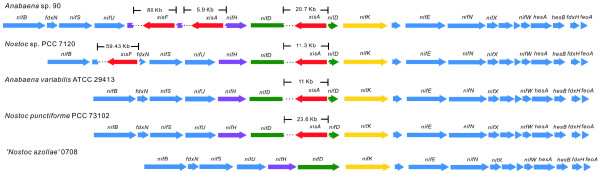
**Comparative illustration of genetic organization of *nif *operon in heterocyst-forming cyanobacteria.** The gene organizations of *nif* operons in heterocyst-forming cyanobacteria are demonstrated in colour as *nifH* in purple, *nifD* in green*, nifK* in yellow and others in blue. Each excision element was indicated by a site-specific recombinase labelled in red and three dots that represent the remaining part of the element. The lengths of the excision elements are shown above

*Anabaena* sp. 90 was grown in the laboratory in nitrogen-free medium and is capable of active nitrogen fixation. The *nif* operon, encoding for the dinitrogenase and dinitrogenase reductase enzyme complexes, was located in chromosome I with conserved gene organization (Figure 9). Three excision elements of 20.7, 5.9 and 80 kb were found within the *nif* operon in *Anabaena* sp. 90. Each element is adjacent to a single site-specific recombinase in the opposite strand that removes the elements during heterocyst development [31]. The 80- and 5.9-kb excision elements that split the *nifH* gene into three parts with sizes of 153, 273 and 444 bp have not been described previously in cyanobacteria. The third element, which commonly occurs in heterocyst-forming cyanobacteria (Figure 9), splits the *nifD* gene into two parts with 1,356 and 147 bp. The *nif* operon spans 122 kb including the three excision elements. The 80-kb element contains one of the prophage remnants. In addition, the fourth 11-kb excision element was detected within the nitrogen-fixation associated *hupL* gene. Surprisingly, the counterparts of *patS* and *hetN*, both involved in pattern formation by preventing neighbouring cells from undergoing heterocyst differentiation [32], could not be detected in this genome. 

### Transporter proteins

The genome shows a remarkable density of genes encoding transporter proteins. There are four porin genes that encode channels for passive nutrient diffusion. A variety of ATP transport systems, often in operons, are present in both chromosomes (see Additional file 1: Table S6) for active uptake of various substrates, such as cations and anions, nucleosides, amino acids, sugars, glycolipids and polyamines. Six copies (three complete, two partial and one disrupted) of the ABC transporter operon *devBCA*, which encode essential exporters for heterocyst envelope formation [33], are present. Two of these are encoded in chromosome II.

### Signal transduction and gene regulation

In all, 153 ORFs, including those carrying insertions, were annotated for signal transduction and regulation. A total of 69 ORFs (see Additional file 1: Table S7), scattered over both chromosomes, were predicted to be involved in two-component signal transduction systems. They include 19 histidine kinases (of which three were pseudogenes), 31 response regulators and 19 hybrid kinases according to their domain composition. These also include five pseudogenes. There are 32 Ser/Thr type protein kinases, six protein phosphatases and other regulation or sensor domain-containing proteins that form the one-component systems that coordinate with the two-component systems. Moreover, all group 1 (*sigA*) and group 2 (*sigB*, *sigC*, *sigD*, *sigE*) sigma factors are present in the *Anabaena* sp. 90 genome. The common group 3 (*sigF*, *sigG*) and one extracytoplasmic function sigma factors were found as well. Four proteins with anti-sigma-factor antagonist domain were also identified. They work together with the sigma factors in regulating various cell processes at the transcriptional level.

### Gas vesicle gene cluster

**Figure 10:**
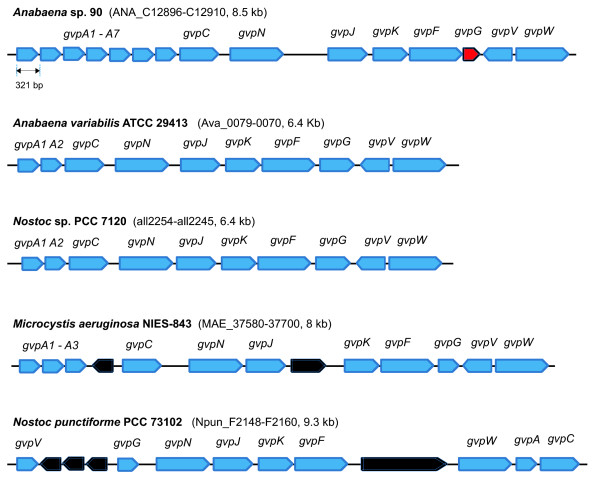
**Genetic organization of cyanobacterial gas vesicle (*gvp*) operons.** The gas vesicle formation genes were indicated in blue. The truncated *gvpG* gene in *Anabaena* sp. 90 was indicated in red. The other genes in the *gvp* operons were indicated in black

An 8.5-kb *gvp* gene operon encoding the building blocks of gas vesicles was located in chromosome I. The operon organization (*gvpA*_*7*_*CNJKFGVW*) is similar to that in other sequenced cyanobacterial strains [34], but with seven tandem *gvpA* genes (Figure 10). A truncated *gvpG* gene was found. This coincided with loss of buoyancy from cells in the present culture, while the original culture showed the buoyant phenotype. 

### Metabolic pathway analysis

We annotated 206 putative metabolic pathways in the genome of *Anabaena* sp. 90 (see Additional file 1: Table S8), in addition to those for bioactive peptide biosynthesis. These pathways are composed of 777 enzymes that catalyse 1,211 enzymatic reactions. However, nearly half of these pathways are incomplete, because 227 (29%) enzyme-encoding genes are missing or were not found. Many of the incomplete pathways are responsible for catabolic processes, such as nutrient degradation, utilization and assimilation, whereas the essential pathways are complete, e.g. amino acid metabolism, photosynthesis and glycolysis. The energy-related pathways have fewer missing enzymes than others. Nearly 40% of the genes in this genome are hypothetical or with unknown functions, due most likely to their low homology to counterparts in model organisms. This may leave some of the enzymes in annotated pathways unrecognized. Moreover, our analysis revealed that some enzyme-encoding genes are pseudogenes with disrupted ORF. For instance, one (ANA_C20606) of the two deoxycytidine triphosphate deaminases, which catalyse the conversion of dCTP to dUTP in the pyrimidine deoxyribonucleotide de novo biosynthesis pathway, was interrupted by an inserted DNA recombinase. In addition, an alcohol dehydrogenase-encoding gene (ANA_C20081) was found with a deletion. This enzyme catalyses the reduction of acetaldehyde to ethanol in fermentation pathways. Three copies of alcohol dehydrogenases were found in this genome in an intact form.

## Discussion

Here, we report the complete genome of *Anabaena* sp. 90, an ecologically important hepatotoxic bloom-forming cyanobacterium. The genome has a multichromosome composition with essential metabolic core genes encoded in the two circular chromosomes. This study was the first to report such a multichromosome composition in the order of Nostocales. Previously, the strain of *Cyanothece* sp. ATCC 51142 was known with two chromosomes, one circular and one linear [35]. We succeeded in completing the genome with high sequence quality, even though it contains a plethora of repetitive mobile genetic elements with diverse sizes (prophage remnants: ~11 kb, ISs: 1–3 kb, MITEs: 100–300 bp) and five nearly identical rRNA operons. A large number of repeats makes genome assembly difficult and cannot be resolved simply by increasing the sequencing depth e.g. using the next-generation low-cost sequencing platforms, due in large part to their short read lengths [36]. As a result, the majority of genomes remain in the ‘draft’ state, including a number of bloom-forming cyanobacteria [17-19]. In this study, we tackled this problem by including data from mate-pair libraries with large inserts [36]. This strategy could be used in sequencing bloom-forming cyanobacterial genomes that are rich in mobile genetic element-derived repeats [16,17,19]. Complete and high quality genomes are crucial to comparative genomics and genome evolution studies [37], pathway reconstruction for metabolic engineering [38] and postgenomic analysis [39-41].

The *Anabaena* sp. 90 genome contains various types of mobile genetic elements, including plasmids, prophage remnants, ISs and MITEs. They are collectively termed as mobile genetic elements, since they are capable of moving within genomes (duplication) and between prokaryotic organisms (horizontal gene transfer) [42]. These elements may have contributed to genome plasticity, genomic rearrangements and most likely the multichromosome composition. ISs are mobile genetic elements transferred within and between species through a cut-and-paste mechanism, which is driven by the internally encoded transposases [43,44]. The percentage of IS elements in *Anabaena* sp. 90 (~2%) was comparable to that in other cyanobacteria, but less than in the bloom-forming hepatotoxin-producing *Microcystis aeruginosa* strains, in which IS elements comprised about 10% of the genomes [45]. Recent genome studies showed that the planktonic cyanobacteria *Cylindrospermopsis raciborskii* CS505 and *Crocosphaera watsonii* WH8501 also contain high numbers of repetitive elements, partly contributed by ISs [19]. MITEs are small mobile sequences that have only terminal inverted repeats [25]. The MITEs found in this study were classified as type II, because there is no evident similarity found with inverted repeats of the ISs in this genome [45]. The mobilization of type I MITEs was hypothesized to be mediated by the transposase of IS that holds the same terminal inverted repeats [46]. This may imply an autonomous mechanism for the movement and duplication of type II MITEs.

Surprisingly, we found that nearly 5% of the gene repertoires are pseudogenes that include not only disrupted transposases but also disrupted ORFs from many other different functional categories (Figure 3). However, the pseudogenes found in the *Anabaena* sp. 90 genome were most likely derived from the transposition activities of ISs and MITEs. Mutations and genome rearrangements induced by transposable elements have also been described in *Microcystis aeruginosa* strains [16,17]. However, an abundance of pseudogenes has rarely been reported in genomes of cyanobacteria, except the endosymbiotic strain ‘*Nostoc azollae’* 0708, which has an extremely high number (31.2%) of pseudogenes that were attributed to the genome erosion process [47]. Here, we accurately labelled a number of disrupted ORFs, many of which could be associated with the transpositions of mobile genetic elements, through detailed manual annotation (Figures 1 and 4). Recent metagenomic data analysis also demonstrated the incidence of genes inactivated by IS transposition, including some with essential functions, within the population of *Synechococcus* strains in hot spring mats [48]. High frequencies of mobile genetic element-derived pseudogenes are likely to be common among transposable element-rich cyanobacteria, especially in strains of bloom-forming genera, but as yet remain undocumented.

*Anabaena* sp. 90 dedicates 5% of its genome to biosynthesis of small peptides, such as hepatotoxic microcystins and the protease inhibitors anabaenopeptins and anabaenopeptilides. Between 3% and 4% of the genomes is typically devoted to the biosynthesis of secondary metabolites among sequenced cyanobacterial genomes [17,18,49]. However, previous works only counted nonribosomal gene clusters and missed the ribosomal biosynthesis pathways. Here, we took into account both ribosomal and nonribosomal gene clusters. Considering the widespread occurrences of ribosomal pathways in cyanobacteria [14,15] and that nonribosomal gene clusters also amount to 3.5% of *Anabaena* sp. 90 genome, it appears that about 5% of the genome is commonly assigned to natural product biosynthesis in cyanobacteria. An additional nonribosomal gene cluster responsible for production of the antifungal compounds hassallidins was discovered during genome assembly, mainly because the gene cluster was inactivated by a deletion event. Moreover, the genome sequence led us to discover the ribosomal production pathway for cyanobactins and their common occurrence in *Anabaena*[29] as well as in other planktonic cyanobacteria [50]. We also identified seven putative bacteriocin gene clusters in the genome. Their end products are presently unknown, but the high number of these types of gene clusters in cyanobacterial genomes [15] suggests that yet new compound families from cyanobacteria await identification and structural determination. To date, bacteriocins have only been identified in *Prochlorococcus marinus* MIT9313 [51].

The toxin microcystin synthetase (*mcy*) gene cluster was shown to be of ancient origin [52]. Previous environmental studies showed the stable presence of mutant cyanobacterial *mcy* gene clusters, which were inactivated both in freshwater samples by ISs [53] and in brackish water by MITEs [54]. Note that the intact hassallidin gene cluster is still retained in parallel cultures of mutant *Anabaena* sp. 90, which has an inactivated anabaenopeptilide synthetase gene cluster [26]. Our results showed that those mutants with an inactive hassallidin biosynthetic pathway prevailed over cells with functional genes in the culture. This may indicate a growth advantage for cells with mutated bioactive compound synthetase gene clusters under laboratory conditions, perhaps due to a lower metabolic burden.

Similarly, we detected a loss of gas vesicles, the subcellular structures responsible for buoyancy, which are essential for bloom-forming planktonic cyanobacteria [3]. This phenotype is derived from the truncated *gvpG* gene in the *gvp* operon (Figure 10). The truncation most probably was selected when the strain was purified (axenic) on solid media [55]. Loss of buoyancy due to rearranged gas vesicle gene clusters by IS transpositions was also previously described in *Microcystis* strains kept in laboratory culture [56].

*Anabaena* sp. 90 has been under continuous culture since 1986 and was introduced into pure culture in 1992 [55]. The mobile genetic elements were probably acquired by the strain prior to this time. Some metabolic properties of *Anabaena* sp. 90 were altered by the activities of these mobile genetic elements. We found mobile element-derived pseudogenes among the genes encoding enzymes for the metabolic pathways of the strain. This may indicate that *Anabaena* sp. 90 has lost genes for metabolic pathways with nonessential functions under laboratory conditions, where optimal, nutrient-rich and competitor-free environments are provided. Our analysis revealed that the laterally acquired mobile genetic elements may have played a role in the process. Perhaps growing cyanobacteria under conditions that reflect those found in nature would reduce genetic changes.

The *Anabaena* sp. 90 genome contains 31 RM systems, which function as microbial defence systems against foreign DNAs [57]. Type I, II, III and IV RM systems were annotated from the *Anabaena* sp. 90 genome, type II restriction enzymes being the most numerous RMs (22). Previously, restriction enzyme activities, such as AflIII, in *Anabaena* sp. 90 were experimentally confirmed [58]. An RM system usually contains a restriction endonuclease that recognizes a specific sequence for cleavage and a DNA methyltransferase that modifies the same sequence and protects it from cleavage [59]. Bacteria that possess multiple RM systems are thought to be virtually impregnable. This seems to hold true for *Anabaena* sp. 90 since it is resistant to genetic manipulation. Over the years we have succeeded in producing only one mutant of this strain [26]. This is a familiar feature with many filamentous cyanobacteria [60], which were proposed more intensively protected by RM systems than unicellular strains [61]. However, this has been contradicted by the frequent occurrence of RM systems in genomes of unicellular toxic bloom-forming *Microcystis aeruginosa* strains [16,17]. To date the two highest numbers of restriction enzymes are found in two planktonic microcystin-producing cyanobacteria, *Anabaena* sp. 90 (this study) and *Microcystis aeruginosa* NIES-843 (Figure 8). This perhaps is a reflection of the ecological or evolutionary pressures exerted on planktonic cyanobacteria. The *Anabaena* sp. 90 genome also contains abundant mobile genetic elements. The question that naturally arises is how these mobile elements have invaded the planktonic cyanobacterial genomes with the presence of pronounced RM systems. The mobile genetic elements are, however, relatively short sequences (see Additional file 1: Tables S3 and S4) and may lack many restriction cleavage sites of restriction endonucleases. For *Anabaena* sp. 90, this may be partly explained by the disrupted genes encoding enzymes in RM systems (see Additional file 1: Table S5). In addition, it is known that RM systems may be inefficient in blocking single-stranded or modified foreign DNA and work only temporarily [62]. However, it was suggested that RM systems may also be mobile genetic elements that cause genome rearrangements [63].

In filamentous cyanobacteria, the cellular processes of photosynthesis and nitrogen fixation are spatially separated and compartmentalized into vegetative cells and heterocysts, respectively [10]. *Anabaena* strains have long been used as model organisms for studying heterocyst development and nitrogen fixation in cyanobacteria [1,64]. The differentiation processes from vegetative cells to heterocysts involve programmed DNA rearrangements at multiple sites in *Anabaena* genomes [65-67]. To date, three known elements, ranging from 11 to 55 kb, have been reported in *nifD*, *fdxN* and *hupL* genes in heterocystous cyanobacteria [67]. Here, for the first time we report the simultaneous presence of four excision elements in the genome of *Anabaena* sp. 90. Moreover, two novel elements for the *nifH* gene were found and one of these is the largest known in size (80 kb) (Figure 9). The *nifH* gene is commonly used to detect nitrogen-fixing organisms in environmental samples. The presence of excision elements is likely to cause problems with detection of *nifH* genes if they occur commonly among heterocystous cyanobacteria. Our results showed that the heterocyst differentiation process in cyanobacteria involves precise genomic splicing over distances as long as 122 kb. The excision elements interrupting *nif* genes in heterocystous cyanobacteria appear to play a role in protecting nitrogenase and hydrogenase from the effects of oxygen during heterocyst development [68]. A compact and conserved *nif* operon was recently discovered without any excision elements in a symbiotic strain ‘*Nostoc azollae*’ 0708 (Figure 9) [47]. This evidence supports the proposed loss of photosynthetic activity in cells of ‘*Nostoc azollae*’ 0708, which may have differentiated only to perform nitrogen fixation for the hosts [47]. A similar *nif* operon without excision elements was presented in the heterocyst-forming cyanobacterium *Cylindrospermopsis raciborskii* CS-505, but lost in its closely related strain *Raphidiopsis brookii* D9 [19]. This may suggest that the growth of these strains is less dependent on the nitrogen fixation process. The presence of multiple copies of *devBCA* operons in *Anabaena* sp. 90 may increase the efficiency of nitrogen fixation, whereas lack of the *patS* and *hetN* genes in *Anabaena* sp. 90 (this study) and *Cylindrospermopsis raciborskii* CS-505 [19] may suggest a new pattern of heterocyst spatial development. Simultaneous inactivation of *patS* and *hetN* genes in *Nostoc* sp. PCC 7120 is known to be lethal, due to overproduction of HetR and heterocysts [69]. Genome mining of *Cylindrospermopsis raciborskii* CS-505 suggested that a protein with the C-terminal pentapeptide RGSGR may take over the function of PatS [19]. Similarly, we also identified an ORF (ANA_C10293) with the C-terminal pentapeptide in *Anabaena* sp. 90, which may play a role as a HetR suppressor.

The 5.3-Mb *Anabaena* sp. 90 genome contains a lower number of genes for signal transduction than the 7.2 Mb-genome of *Nostoc* PCC 7120 [64] and nearly 10-Mb genome of *Nostoc punctiforme*[70]. However, the number of signal transduction pathways is positively correlated with genome size. It is also well known that soil microbes such as *Nostoc punctiforme* invest more heavily in sensing changes in environmental conditions than organisms living in more stable aquatic environments. Usually, a one-to-one relationship exists between the cyanobacterial sensors (histidine kinases) and response regulators [71], but in *Anabaena* sp. 90 the ratio is lower, indicating either integration of multiple signalling pathways or perhaps loss of sensoring systems when grown in protected laboratory environments.

## Conclusions

This study gives a snapshot of the *Anabaena* sp. 90 genome. It shows a high potential of genetic variation by virtue of the occupation of a wide range of mobile genetic elements. Our results indicated that mobile genetic element-imposed selective pressure led to genome adaption to the strain by trimming nonessential genes and pathways during cultivation in the laboratory. In addition, due to the array of biosynthesis gene clusters for multiple peptides in *Anabaena* sp. 90, the complete sequence provides a valuable research subject in studying the regulation of natural product biosynthesis, which may have potential pharmaceutical and biotechnology applications.

## Methods

### Strain isolation and culture

*Anabaena* sp. 90 was isolated as a microcystin producer in 1986 from Lake Vesijärvi, Finland [72]. The axenic culture was originally purified from a single filament that was placed over a solid medium and then has been continuously maintained at the University of Helsinki cyanobacterial culture collection in Z8 nitrogen-free medium at room temperature (20–23°C) with continuous illumination of 10–20 μmol m^-2^ s^-1^[8]. The phylogeny of this strain was previously published [73].

### DNA extraction and genomic library construction

The DNA extraction was described earlier [26]. Three sizes of genomic libraries were utilized for end sequencing. The large insert library was a cosmid library with an insert size of approximately 40 kb [26]. Two shotgun libraries with 2-kb and 6-kb inserts ligated into the pUC18 plasmid vector were constructed using standard protocols.

### Genome sequencing, assembly and finishing

All reads were generated from clone ends sequencing by the Sanger sequencing platforms Megabase 1000 (GE Healthcare, Buckinghamshire, UK) and ABI 3730 (Applied Biosystems, CA, USA). An initial 9.9X sequencing data was sequenced from a combination of 2%, 8% and 90% of the reads from the 40-kb, 6-kb and 2-kb libraries, respectively. The Phred/Phrap/Consed software package [74] was used for genome assembly and gap closure according to the paired ends from the large-insert (6 and 40 kb) libraries. The remaining physical gaps that were derived from the unclonable regions were linked through combinatorial multiplex PCR screening of primers designed from the contig ends. Autofinish [75] was used for guiding, either by clone-end resequencing or primer walking over the clones or PCR products to attain the standard that each base was covered by at least two independent high-quality reads and with a Phred [76,77] quality value ≥ Q40. Large repetitive regions (i.e. RNA operons, prophage remnants) were resolved by primer-walking over long PCR products amplified from the corresponding regions. In all, 119316 reads were produced, which amounted to a final sequencing depth of 12.5X.

### Genome annotation and analysis

#### Gene finding and function assignment

ORFs were initially predicted by Glimmer 3.02 [78] with a threshold of 100 bp. The intergenic regions were subjected to blastx searching [79] against the nonredundant database for unrecognized ORFs. All predicted genes were translated into amino acid sequences for homologue searches with the InterPro [80], Cluster of Orthologous Groups [81] and nonredundant databases. Functional assignments and start sites for each ORF were determined manually by combining the search results from these sources. Transfer RNA genes were predicted with tRNAscan-SE [82] and rRNA genes were located through homologue searches. The annotated proteins were further assigned to functional groups according to the Comprehensive Microbial Resource role category [20]. The putative bacteriocin gene clusters were classified, according to methods described previously [15]. Hypothetical proteins were defined as conserved if they had at least 30 homologues with full-length matching in other genomes, while unique hypothetical proteins had no full-length matching in other genomes.

#### Pseudogenes

The pseudogenes (see Additional file 1: Table S2) were examined manually using Artemis [83] for frameshift and premature stop codons, as well as the boundaries of the truncation, deletion and insertion. The boundaries of truncated pseudogenes were determined through iterative BLAST searches for the surrounding regions. The pseudogenes were assigned a function according to the hits of the homologue search with significant similarity.

#### IS and MITE

The ISs were identified and classified using the ISfinder database [24]. Fifteen MITEs were originally discovered as insertions from interrupted pseudogenes. Additional MITEs were found by blastn searching the 15 insertions against the complete genome sequence (*E* < 1e-5). Fragmented MITEs with over 50% coverage were counted as partial. The secondary structures of the MITEs were predicted using the RNAfold webserver [84].

#### RM systems

A protein database of RM system genes was locally prepared by collecting data from the REBASE [85]. All annotated proteins initially searched against this database, and protein hits of blastP (*E* < 1e-4) were chosen as candidates for manual checking. RM systems were confirmed with the associated presence of restriction enzymes, DNA methylase and other RM genes or domains. Separate RM genes were kept while the *E* value was 1e-10 or less. The disrupted RM genes were reconfirmed by manually inspecting the alignment of the remaining parts.

#### Metabolic pathway analysis

A pathway/genome database of *Anabaena* sp. 90 was constructed using Pathway Tools [86] from the annotation. Pathway Hole Filler [87] was further used for retrieving the missing enzymes.

### Hassallidin

#### DNA extraction and PCR

Both *Anabaena* sp. 90 and a knockout mutant constructed in 1999 [26] were grown as previously described. Genomic DNA from the two *Anabaena* strains was extracted using either the DNeasy® Plant Mini Kit (Qiagen Gmbh, Hilden, Germany) or the E.Z.N.A™ SP Plant DNA Mini Kit (Omega Bio-Tek Inc., Norcross, GA, USA). PCR amplifications of DNA from *Anabaena* were performed in iCycler (Bio-Rad, Hercules, CA, USA) using the primers hasV-fw (5-TCTAGATGGTTGGAGTGTGGC-3) and hasV-rev (5-AGGATGCGGTAGCTTTGAGGAGGCG-3), which were designed to amplify across the site of deletion in the *hasV* gene. The primer pairs a1 (5-TGGTAACAGGTAACGTAATTAAAAC-3) and hasV-rev, and y1 (5-ATCAGTAGTTTCGGGTCTGG-3) and hasV-fw were designed to allow specific detection of deletion and full-length versions of *hasV,* respectively*.* Each PCR was carried out in 1 × DyNAzyme^TM^ buffer (Tris–HCl, MgCl_2_, KCl, Triton® X-100) (Finnzymes, Espoo, Finland) with 100 μM of each dNTP (dNTP mix, 10 mM, Finnzymes), 0.25 μM of each primer (Sigma Genosys, Sigma-Aldrich, St. Louis, MO, USA), 0.4 U DyNAzyme II DNA polymerase (F-501S, 2 U/μl, Finnzymes) and 10–170 ng of template DNA in a final volume of 20 μl. The following PCR thermocycle was used to amplify the *hasV* gene: initial denaturation at 94°C for 3 min, 32 cycles of 94°C for 30 s, 57°C for 30 s and 72°C for 1.5 min, and a final extension at 72°C for 10 min. For detection of the deletion, a similar PCR programme was used, with the exception of annealing at 64°C for 30 s and elongation at 72°C for 1 min.

#### Chemical analysis

Cells of the cyanobacterial strains were collected from the 20–40-ml cultures by centrifugation at 7000 × g for 7 min and freeze-dried (Maxi dry plus, Heto-Holten A/S, Allerød, Denmark). The dried cells were extracted with 1 ml of methanol in 2-ml plastic tubes containing glass beads (Cell Disruption Media, 0.5-mm glass beads, Scientific Industries Inc., Bohemia, NY, USA) using a FastPrep cell disrupter (FP120, Bio101, Thermo Electron Corporation, Qbiogene, Inc., CA, USA) for 10 s at a speed of 6.5 m s^-1^. The extracts were centrifuged at 10 000 × g 5 min prior to LC/MS analysis. The LC/MS analyses of extracts were carried out in an Agilent 1100 Series LC/MSD Trap XCT Plus System (Agilent Technologies, CA, USA) using a Phenomenex Luna C18(2) LC column (100 mm × 4.6 mm, particle size 5 μm, 100 Å; Phenomenex, CA, USA). The column was protected with a C18 precolumn (4 mm × 2 mm; Phenomenex). The LC/MS parameters were optimized with hassallidin ion m/z 1862 with negative mode of polarity. The mobile phase consisted of 0.1% aqueous formic acid (50% solution in water; Fluka, Sigma Aldrich, Steinheim, Germany) (solvent A) and acetonitrile (Chromasolv® for LC/MS, Fluka, Sigma-Aldrich, Steinheim) (solvent B). A gradient (solvent B) from 10% to 100% was run over 30 min at a flow rate of 0.15 ml min^-1^ with 5-μl injection at 40°C. Hassallidins were detected with MS using electrospray ionization set in the positive ion mode. The nebulizer gas (N_2_) pressure was 35.0 pounds per square inch (psi), the drying gas flow rate was 8.0 l min^-1^ and the temperature was 350°C. The capillary voltage was set to 3650 V, and the capillary offset value was 250 V. A skimmer potential of 58.0 V and a trap drive value of 112.0 was used. The spectra were recorded at a scanning range of 100–2200 m/z on average of three spectra using an ion charge control (ICC). Identification of hassallidins was based on the similarities between the spectra of the extracts analysed and known hassallidins A and B (Alexis Biochemicals, Ezno Life Science Inc., Lausen, Switzerland). The structures of the hassallidins were identified by fragmentation analysis MS^n^ (n = 1–3) using the SmartFrag function.

### Accession number

The complete annotated genome sequence was submitted to GenBank under accession number [GenBank: CP003284 (ChromosomeI), CP003285 (ChromosomeII), CP003286 (PlasmidA), CP003287 (PlasmidB), CP003288 (PlasmidC)].

## Abbreviations

bp: Base pair; kb: Kilobase pairs; ORF: Open reading frame; IS: Insertion sequence; MITE: Miniature inverted-repeat transposable element; RM: Restriction-modification; CRISPR: Clustered regularly interspaced short palindromic repeat.

## Competing interests

The authors declare that they have no competing interests.

## Authors’ contributions

HW carried out the genome assembly, gap closure and detailed genome analysis. ZL and BL were responsible for sequencing of the genome. HW, LR, CL, ARY and KR manually annotated the genome. JV, LR, DPF analysed the hassallidin gene cluster and JJ conducted the chemical analysis. HW, KS, DPF, ARY, LR, CL, JV and JJ drafted the manuscript. KS conceived of the study, and participated in its design and coordination. All authors read and approved the final manuscript.

## Supplementary Material

Additional file 1 **Table S1.** Hypothetical proteins in the *Anabaena* sp. 90 genome. Hypothetical proteins with disrupted ORF are labelled in yellow. **Table S2.** Pseudogenes annotated from the *Anabaena* sp. 90 genome. **Table S3.** Summary of ISs identified in the *Anabaena* sp. 90 genome. **Table S4.** List of MITEs discovered in the *Anabaena* sp. 90 genome. The MITEs that disrupted chromosome ORFs by insertion are labelled in green. **Table S5.** RM system genes in the *Anabaena* sp. 90 genome. **Table S6.** Summary of transporter proteins in the *Anabaena* sp. 90 genome. **Table S7.** Two-component system genes annotated from the *Anabaena* sp. 90 genome. The two-component genes were classified as HK, RR and HR, representing histidine kinase, response regulator, and hybrid kinase, respectively. Pseudogenes with disrupted ORF are labelled in yellow. **Table S8.** Predicted metabolic pathways of *Anabaena* sp. 90. Click here for file
